# Cystitis and Utipro^®^ Plus: Real-World Evidence

**DOI:** 10.3390/healthcare11182564

**Published:** 2023-09-16

**Authors:** Karel Kostev, Tommaso Cai

**Affiliations:** 1 Epidemiology, IQVIA S.L., 60549 Frankfurt am Main, Germany; 2 Department of Gynecology and Obstetrics, University Hospital Marburg, Philipps-University Marburg, 35043 Marburg, Germany; 3 Department of Urology, Santa Chiara Hospital, 38122 Trento, Italy; ktommy@libero.it; 4 Institute of Clinical Medicine, University of Oslo, 0318 Oslo, Norway

**Keywords:** medical device, xyloglucan, urinary tract infection, real-world studies

## Abstract

Background: The emergence of drug resistance in the etiological agents of uncomplicated urinary tract infections (UTIs) emphasizes the need to shift the paradigm towards alternative therapeutic strategies. The objective of the present study was to evaluate the use of a medical device containing xyloglucan, hibiscus, and propolis for reducing UTI symptomatic episodes, antibiotic prescription, and days of sick leave. Materials and Methods: It used retrospective, cross-sectional study data provided by office-based physicians from Germany (Disease Analyzer, IQVIA database), including those on 3586 patients with a diagnosis of UTI treated with Utipro^®^ Plus (Noventure, Barcelona, Spain) from January 2015 to December 2020. Results: The majority of patients were women (94.2%) and had a mean age (standard deviation, SD) of 57.7 years (19.0). Within 12 months after the prescription and compared to the 12 months before, it was observed that there was a reduction in the proportion of patients with at least one UTI diagnosis (from 79.4% to 36.4%, *p* < 0.001), in antibiotic prescriptions (from 33.5% to 22.1%, *p* < 0.001), and in the proportion of patients with at least one day of sick leave (from 4.1% to 2.7%). Conclusions: The use of Utipro^®^ Plus is able to decrease UTI recurrence and can lead to the reduction of antibiotic prescriptions and disease burden in individuals affected by uncomplicated cystitis.

## 1. Introduction

Urinary tract infections (UTIs) are the most frequently diagnosed type of infection in the outpatient setting, mostly affecting women [1,2]. These infections can range in severity from uncomplicated UTIs (e.g., uncomplicated cystitis) to urosepsis [3]. Every second woman experiences at least one UTI in her lifetime, but UTI prevalence differs considerably by age group [4]. The main etiological agent of UTI is Escherichia coli (*E. coli*) [5]; however, the pathogenic microorganisms of UTI are various [6]. UTIs are typically treated with antibiotics, but there is growing concerned about the rising prevalence of resistance among Gram-negative uropathogens such as *E. coli* [7]. Moreover, the burden of recurrent UTIs is considerable and has both personal and societal aspects that include the clinical and economic burden of the illness and psychological effects that have a negative impact on the quality of life (QoL) [8]. UTI burden, together with the dramatic increase in the spread of multidrug-resistant microorganisms, has urged the interest in and research focused on the non-antibiotic prophylaxis of UTIs, specifically the use of phytotherapy and nutraceuticals as feasible and effective alternative approaches [9,10,11]. These methods aim to reduce the use of antibiotics and the frequency of symptomatic recurrences [12].

Utipro^®^ Plus (Noventure SL, Barcelona, Spain) is a non-pharmacological medical device that was developed to prevent and control UTIs caused by *E. coli* and other Gram-negative uropathogens. The main components of Utipro^®^ Plus are xyloglucan (soluble hemicellulose) and gelatin, which, when combined with propolis and extracts of Hibiscus sabdariffa, work to prevent bacteria from adhering to the mucosa of the intestine [13]. This mechanical action is crucial in minimizing the adhesion and proliferation of uropathogens in the intestinal lumen and avoids their migration from the urethra to the bladder [14]. In addition to this, Utipro^®^ Plus also acidifies urine pH (using hibiscus and propolis) to control bacterial growth and reduce local urinary symptoms associated with the inflammation of the bladder mucosa [15,16]. Utipro^®^ Plus has demonstrated efficacy in several clinical trials both for acute uncomplicated UTIs and in the long-term prevention of recurring UTIs [17,18]. However, data from real-world studies showing the effectiveness of Utipro^®^ Plus are limited. The present study aims to gain insights into both the impact of Utipro^®^ Plus prescriptions on the reduction of UTI symptomatic episodes as well as the reduction of antibiotic therapy and days of sick leave.

## 2. Materials and Methods

### 2.1. Data Source

This was a retrospective, cross-sectional study based on Disease Analyzer (DA) IQVIA database, which contains case-based information provided by office-based physicians (both general practitioners [GPs] and specialists) in Germany. This platform collected information from more than 10 million patients in the period lasting from 2009 to 2021 on demographics, drug prescriptions, concomitant medication, comorbid conditions, sick leave, and hospital referrals. The information was provided by almost 3000 office-based physicians, representing approximately 3% of all German practices (DA status date: September 2021). Practices can be classified into 10 categories according to the medical profession of the physician in question. The sample of practices included is geographically representative for Germany, covering eight large German regions. The data analyses will only take into account data from facilities that have continuously provided data to the DA panel in the past [19].

### 2.2. Ethical Aspects

German law allows the use of anonymous de-identified electronic medical records for research purposes under certain conditions. According to this legislation, it is not necessary to obtain informed consent from patients or approval from a medical ethics committee for this type of observational study, which contains no directly identifiable data. Therefore, no waiver of ethical approval was obtained from an Institutional Review Board (IRB) or ethics committee. The company and the involved authors had no access to any identifying information at any moment during the analysis of the data.

### 2.3. Study Population

This study included patients treated by general practitioners (GPs), gynecologists and urologists with a prescription of Utipro^®^ Plus from January 2015 to December 2020. Utipro Plus^®^ must be taken orally via 2 capsules per day (1 capsule every 12 h) for five days. For the prevention of recurrence, one capsule must be taken per day for at least 15 consecutive days per month. If required, the treatment can be repeated. Patients had had at least 12 months of observation time prior to the first prescription, as well as 12 months of follow-up time after the first prescription and a diagnosis of urinary tract infection (ICD-10: N39.0) or cystitis (ICD-10: N30.0, N30.8, N30.9) within 12 months before the Utipro^®^ Plus prescription or earlier. Patients with missing data on age or sex were excluded. No more inclusion and exclusion clinical criteria were used in order to obtain a real-world experience.

### 2.4. Outcomes, Co-Variables and Statistical Methods

The outcomes considered in this study were: (1) number of UTI diagnoses within 12 months after the Utipro^®^ Plus prescription compared to 12 months prior to Utipro^®^ Plus prescription, (2) number of antibiotic prescriptions within 12 months after the Utipro^®^ Plus prescription compared to 12 months before Utipro^®^ Plus prescription, and (3) number of days of sick leave due to UTI within 12 months after the Utipro^®^ Plus prescription compared to 12 months prior to Utipro^®^ Plus prescription. For each patient, the time point of the first Utipro^®^ Plus prescription was considered the index date.

Descriptive statistics (involving mean value and standard deviation) were performed for continuous variables and the total number of patients (N) and the relative frequencies (%) were obtained for the categorical parameters. Demographic variables included age as a continuous variable, including age groups 18–30, 31–40, 41–50, 51–60, 61–70, and >70 years; sex; and health insurance coverage (private or statutory). Co-diagnoses documented within 12 months before or on the index date included hypertension (I10), diabetes mellitus (E10–E14), obesity (E66), lipid metabolism disorders (E78), ischemic heart diseases (I20–I25), heart failure (I50), stroke/TIA (I63, I64, G45), renal failure (N18, N19), Parkinson’s Disease (G21,G22), dementia (F00–F03, G30), and chronic bronchitis or chronic obstructive pulmonary disease (COPD) (J42–J44).

Differences in the outcomes before vs. after the Utipro^®^ Plus prescription were first descriptively tested using Wilcoxon tests for paired samples. These comparisons were performed separately for women, men, and defined age groups. *p*-values < 0.05 were considered statistically significant. Analyses were carried out using SAS version 9.4 (SAS institute, Cary, NC, USA).

## 3. Results

### 3.1. Study Population

**Figure 1:**
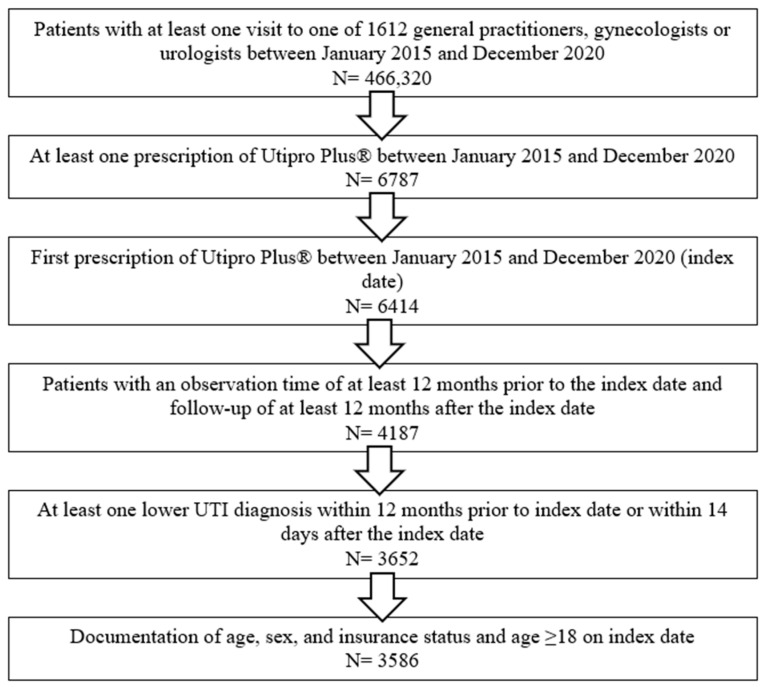
Flow-chart describing the selection procedure of the patients included in this study.

A total of 3586 patients were included in this study. Patients were selected and included in this study according to the flow-chart described in Figure 1. Patient characteristics are displayed in Table 1.

Patients were, on average, 57.7 years old [standard deviation (SD): 19.0], and the majority were women (94.2%). Most of the patients (89.2%) were being treated by GPs, and only 6.4% were treated by gynaecologists and 4.4% by urologists. Statutory health insurance was predominant in comparison with private health insurance (87.1% versus 12.9%). The prevalence of chronic disorders was low: 24.3% of the patients included had hypertension, 12.3% had lipid metabolism disorders, and 8.5% had diabetes. Other diseases such as dementia or Parkinson’s disease had been diagnosed in up to 1% of the study population.

### 3.2. UTI Diagnoses

**Figure 2:**
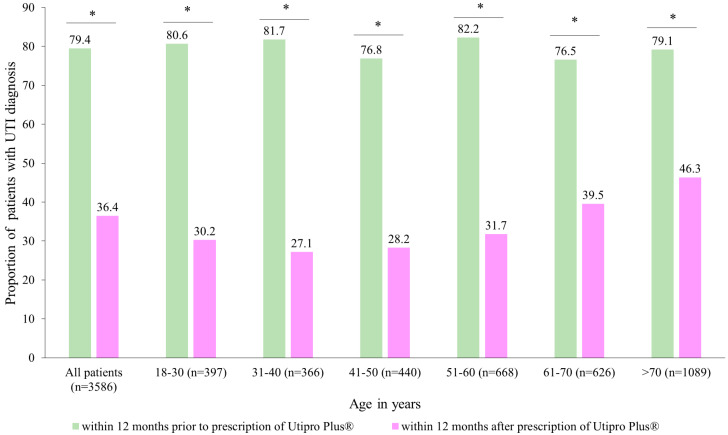
Number of patients with UTI diagnoses within 12 months after the prescription versus within 12 months prior to the prescription of Utipro^®^ Plus. * *p* < 0.05.

Figure 2 describes the proportion of patients with at least one UTI diagnosis in two time periods. After the prescription of Utipro^®^ Plus, the proportion of patients with at least one UTI diagnosis decreased from 79.4% to 36.4% (*p* < 0.001). Similar changes were observed in each age group and in both women and men (Figure 2).

Moreover, it was observed that there was a decrease from 1.3 to 0.7 UTI diagnoses per patient at 12 months after the index date, with a resulting difference of 0.6 diagnoses per patient (*p* < 0.001) (Table 2). The strongest decrease (0.8 diagnoses per patient) was observed in the age group of 31–40 years, and the weakest decrease (0.5 diagnoses) in the age group > 70, with both results being highly significant (*p* < 0.001).

### 3.3. Antibiotic Prescriptions

**Figure 3:**
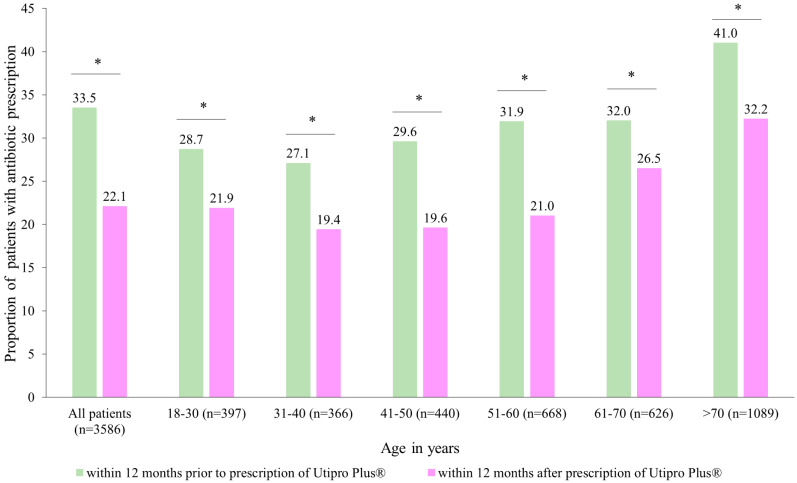
Number of patients with antibiotic prescriptions within 12 months after the prescription versus within 12 months prior to the prescription of Utipro^®^ Plus. * *p* < 0.05.

After the prescription of Utipro^®^ Plus, the proportion of patients with at least one antibiotic prescription decreased from 33.5% to 22.1% (*p* < 0.001) (Figure 3). Significant changes were observed in each age group (Figure 3).

Similar results were obtained in both women (reduction from 33.7% to 25.6%) and men (reduction from 30.6% to 17.2%). Additionally, a significant decrease from 0.5 antibiotic prescriptions was observed per patient 12 months before the index date to 0.4 antibiotic prescriptions after the index date (Table 3). These results lead to a difference of 0.11 antibiotic prescriptions per patient (*p* < 0.001). The decrease in antibiotic prescriptions was greater in men than in women (0.19 vs. 0.11 antibiotic prescriptions per patient). The weakest decrease was observed in patients aged 61–70 years (0.06 antibiotic prescriptions per patient); however, in all subgroups, changes were significant (Table 3).

### 3.4. Days of Sick Leave (DSL)

**Figure 4:**
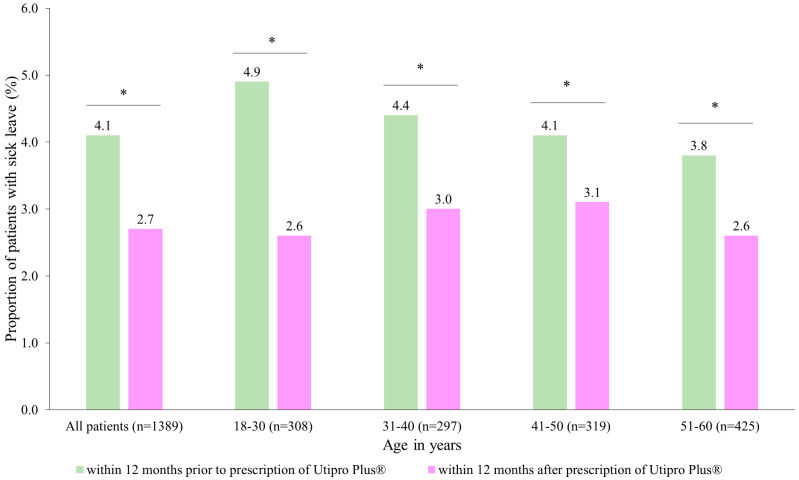
Number of patients with at least one day of sick leave (DLS) within 12 months after the prescription versus within 12 months prior to the prescription of Utipro^®^ Plus * *p* < 0.05.

After the prescription of Utipro^®^ Plus, the proportion of patients with at least one day of sick leave decreased from 4.1% to 2.7% (Figure 4). Similar changes were observed in each age group. No patients were found in the age group of 61–70 years (Figure 4).

When data are analyzed by gender, it is observed that there was a significant difference in DSL in women (from 4.1 to 2.7, *p* < 0.05) but not in men (3.7 DSL in both periods, *p* > 0.5) Additionally, there was a significant decrease from 0.19 DSL per patient at 12 months before the index date to 0.10 DSL per patient at 12 months after the index date, with a resulting difference of 0.09 DSL per patient (*p* = 0.023) (Table 4). In subgroup analyses, the decrease was significant in women only (0.09 DSL, *p* = 0.019) (Table 4).

## 4. Discussion

UTIs are a worldwide health issue associated with a decrease in the quality of life of patients and a significant clinical and economic burden [20]. Their pathogenesis begins with contamination with uropathogens that adhere to epithelial and urothelial cells, migrate to the bladder, and colonize and get internalized by urothelial cells with the subsequent formation of intracellular bacterial communities (IBCs). This adherence depends on a complex host–pathogen interaction, and once internalized, bacteria can rapidly replicate within urothelial cells, forming communities that can evade host defense mechanisms, resulting in quiescent intracellular reservoirs [21]. Chinese herbs, such as Huang Lian (*Coptis chinensis*), have shown both inhibitory activity against a number of uropathogenic bacteria as well as anti-inflammatory effects, while others like Compound Salvia Plebeia Granules help reduce *E. coli* adherence to the bladder’s urothelial cells. In the case of cranberry, it has been proposed that its fructose might have a role in inhibiting the adherence of *E. coli* with type 1 fimbriae to urothelial cell receptors, while its containing proanthocyanidins can affect adherence in P fimbriated *E. coli*. Finally, in the case of probiotics, lactobacilli are thought to compete with uropathogens for vaginal epithelial adhesion receptors, leading to a prevention of colonization by uropathogenic organisms [21]. In addition, the increase in antibiotic resistance [22] and the role of non-antibiotic oral supplements in reducing UTI symptomatic episodes and the use of antibiotics are increasing the interest among physicians and researchers in the use of medical devices containing phytotherapy and nutraceutical compounds [9,10,11]. The current EAU Guidelines for urological infections state that the efficacy of herbal products such as cranberry products or herbal/plant extracts remains unclear, but clinicians can recommend their prescription in combination with antimicrobial therapies for the prevention of UTIs based on their favorable benefit-to-harm ratio [23]. Utipro^®^ Plus, a medical device containing xyloglucan, hibiscus, and propolis, has been used in the management of recurrent UTIs. However, data from real-world studies are limited. In this study, we analyzed, for the first time, the impact of Utipro^®^ Plus prescriptions on reducing the number of UTI symptomatic episodes, antibiotic prescription, and days of sick leave. We observed a decrease in UTI diagnosis, after the prescription of Utipro^®^ Plus, from 79.4% to 36.4% (*p* < 0.001), with this reduction being similar in all age groups and genders (Figure 2, Table 2). The efficacy of this medical device in reducing the number of recurrent UTIs and the severity and frequency of symptomatic UTIs have been demonstrated in clinical trials and prospective studies [15,16,17,18,24,25,26,27]. Costache et al. [27] conducted a randomized, double-blind, multicenter phase IV clinical trial involving 40 women with uncomplicated UTI symptoms. The women received either the medical device or a placebo as an adjuvant therapy to antimicrobial treatment. The authors found that by day 11, the medical device reduced culture positivity (mostly caused by *E. coli*) to 0%, compared to the culture positivity value of 45% for the placebo group. UTI recurrence occurred in 15% and 70% of patients who received the medical device and placebo, respectively, by day 76. Patients who received the medical device also experienced a significant reduction in urinary incontinence and urgency of micturition compared to those who received the placebo. In a review and meta-analysis, Cai et al. found a statistically significant difference in UTI clinical or microbiological resolution between the use of the medical device and the comparator (three randomized clinical trials; 178 patients; OR: 0.13; 95% CI: 0.05–0.33; *p* < 0.0001). Moreover, no clinically significant adverse effects were reported in their studies [28]. In an observational prospective study by Cai et al. [18], 61 women with recurrent UTIs were given the medical device for six months. At the 1-, 3-, and 6-month follow-up visits, 67.2%, 77.0%, and 83.6% of women reported significant improvements in clinical symptoms and the quality of life, respectively. Furthermore, only 11.4% of women experienced at least one symptomatic episode of recurrent UTI requiring antibiotics during the study period. In our previous observational and prospective study, 81.6% of women treated with Utipro^®^ Plus did not require an additional consultation within seven days after the initial one. In addition, among those who did not receive concomitant medication (mostly antibiotics), 89.5% did not require additional consultation [29].

Compared to data published about herbal medicines with an adjuvant role, Utipro^®^ Plus is still superior in reducing UTI incidence. In a meta-analysis by Xia et al. [30], it was observed that the intake of cranberry-based products can significantly reduce UTI incidence in susceptible populations (risk ratio (RR) = 0.70; *p* < 0.01), with relative risk reductions of 32%, 45%, and 51% in women, children, and patients using indwelling catheters, respectively. In a randomized trial comparing *Rosa canina* capsules vs. placebo among 400 women, the results showed that among participants in the intervention group, UTI incidence was significantly lower between the 7th and 10th days (odds ratio = 0.22 [95% CI: 0.07, 0.67], *p* = 0.006) and on the 20th day (odds ratio = 0.32 [95% CI: 0.14, 0.75], *p* = 0.008). However, no statistically significant differences were observed between both groups regarding the incidence of cystitis (*p* > 0.05) [31]. In the case of Chinese herbal products, a meta-analysis has described that in comparison to antibiotics, they have shown a higher rate of effectiveness for acute UTI (relative risk [RR] 1.21, 95% CI: 1.11, 33) and reduced recurrent UTI rates (RR 0.28, 95% CI: 0.09, 0.82). In addition, these herbal products vs. antibiotics alone have led to a higher rate of effectiveness for acute UTI (RR 1.24, 95% CI: 1.04, 1.47), resulting in lower rates of recurrent infection six months after the study (RR 0.53, 95% CI: 0.35, 0.80) [32].

Interestingly, in this study, we observed a reduction in antibiotic use after the prescription of Utipro^®^ Plus, with the proportion of patients with at least one antibiotic treatment decreasing from 33.5% to 22.1% (*p* < 0.001) (Figure 3). These results are in accordance with the literature, where Utipro^®^ Plus appears to have an important and valuable role in everyday clinical practice as a non-antimicrobial option to control and prevent UTI and in the reduction of antibiotic use [13]. Furthermore, we observed a decrease in the proportion of patients with at least one DSL from 4.1% to 2.7% after the prescription of Utipro^®^ Plus (Figure 4). These results are important since are associated with improvements in the quality of life and reducing the disease burden.

Several limitations could be identified in this study. The retrospective nature of the study can be considered a limitation due to the validity and completeness of the data. Moreover, the DA database has some important limitations that should be mentioned. First, assessments rely on ICD codes entered by GPs, urologists, and gynecologists. Second, diagnosis codes do not allow the separation of the severity stages of the diseases or the outcomes. Third, the results of quality-of-life, dosage, and adherence assessments are not available. Also, other variables that can influence UTI reduction were not available, and so, further analysis should be performed. Fourth, patients do not need prescriptions from physicians to buy herbal medicines, which are over-the-counter drugs. The database does not include data on the use of herbal medicines that patients buy without prescriptions.

## 5. Conclusions

Utipro^®^ Plus prescription was significantly associated with a reduced recurrence of UTIs, antibiotic prescriptions, and DSL, documented within 12 months after the Utipro^®^ Plus prescription compared to the same period before the Utipro^®^ Plus prescription. These findings provide additional evidence for the advantage of Utipro^®^ Plus use as a supplementary therapy alongside primary antimicrobial agents in the management of UTIs in adult patients.

## Figures and Tables

**Table 1:** Sociodemographic and clinical characteristics of patients included in the study.

Variable	Patients Treated with Utipro^®^ Plus (n = 3586)
Age mean (SD)	57.7 (19.0)
Age 18–30 years, n (%)	397 (11.1)
Age 31–40 years, n (%)	366 (10.2)
Age 41–50 years, n (%)	440 (12.3)
Age 51–60 years, n (%)	668 (18.6)
Age 61–70 years, n (%)	626 (17.5)
Age > 70 years, n (%)	1089 (30.4)
Gender, n (%)	
Female	3377 (94.2)
Male	209 (5.8)
Insurance, n (%)	
Private health insurance	461 (12.9)
Statutory health insurance	3125 (87.1)
Comorbidities, n (%)	
Hypertension	872 (24.3)
Lipid metabolism disorders	440 (12.3)
Diabetes	304 (8.5)
Obesity	122 (3.4)
Ischemic heart diseases	178 (5.0)
COPD	152 (4.2)
Renal failure	111 (3.1)
Heart failure	92 (2.6)
Ischemic stroke	52 (1.5)
Dementia	36(1.0)
Parkinson’s disease	11 (0.3)

SD: standard deviation; COPD: chronic obstructive pulmonary disease.

**Table 2:** Changes in the mean number of diagnoses per patient within 12 months after the prescription of Utipro^®^ Plus versus within 12 months prior to the prescription of Utipro^®^ Plus.

Variable	Mean Number of UTI Diagnoses per Patient at 12 Months Prior to Prescription Mean (SD)	Mean Number of UTI Diagnoses per Patient at 12 Months after Prescription Mean (SD)	Difference in Number of UTI Diagnoses (95% CI)	*p* Value *
All patients	1.3 (1.4)	0.7 (1.3)	0.6 (0.6–0.7)	<0.001
Age 18–30 years	1.2 (1.1)	0.5 (0.9)	0.7 (0.6–0.8)	<0.001
Age 31–40 years	1.2 (1.0)	0.4 (0.7)	0.8 (0.7–0.9)	<0.001
Age 41–50 years	1.2 (1.1)	0.5 (1.2)	0,6 (0.5–0.8)	<0.001
Age 51–60 years	1.3 (1.2)	0.5 (1.0)	0.8 (0.6–0.9)	<0.001
Age 61–70 years	1.3 (1.3)	0.7 (1.2)	0.5 (0.4–0.6)	<0.001
Age > 70 years	1.6 (1.8)	1.1 (1.8)	0.5 (0.4–0.6)	<0.001
Women	1.3 (1.4)	0.7 (1.3)	0.6 (0.6–0.7)	<0.001
Men	1.2 (1.2)	0.6 (1.5)	0.5 (0.4–0.7)	<0.001

SD: standard deviation; CI: confidence intervals; UTI: uncomplicated urinary tract infection. * Wilcoxon test for paired samples.

**Table 3:** Changes in the mean number of antibiotic prescriptions per patient within 12 months after the prescription of Utipro^®^ Plus versus within 12 months prior to the prescription of Utipro^®^ Plus.

Variable	Mean Number of Antibiotic Prescriptions per Patient at 12 Months Prior to Prescription (SD)	Mean Number of Antibiotic Prescriptions per Patient at 12 Months after Prescription (SD)	Difference in Number of Antibiotic Prescriptions (95% CI)	*p* Value *
All patients	0.5 (0.9)	0.4 (0.9)	0.11 (0.08–0.14)	<0.001
Age 18–30 years	0.4 (0.7)	0.3 (0.6)	0.11 (0.02–0.19)	0.024
Age 31–40 years	0.4 (0.7)	0.3 (0.6)	0.11 (0.02–0.19)	0.005
Age 41–50 years	0.4 (0.9)	0.3 (0.7)	0.14 (0.05–0.22)	<0.001
Age 51–60 years	0.4 (0.8)	0.3 (0.6)	0.17 (0.09–0.23)	<0.001
Age 61–70 years	0.5 (0.9)	0.4 (0.8)	0.06 (0.02–0.14)	0.026
Age > 70 years	0.7 (1.1)	0.6 (1.2)	0.10 (0.02–0.18)	<0.001
Women	0.5 (0.9)	0.4 (0.9)	0.11 (0.07–0.14)	<0.001
Men	0.4 (0.7)	0.2 (0.5)	0.19 (0.07–0.29)	<0.001

SD: standard deviation; CI: confidence intervals; UTI: uncomplicated urinary tract infection. * Wilcoxon test for paired samples.

**Table 4:** Changes in the mean number of sick-leave days per patient within 12 months after the prescription of Utipro^®^ Plus versus within 12 months prior to the prescription of Utipro^®^ Plus.

Variable	Number of Days on Sick-Leave per Patient in 12 Months Prior to Prescription (Mean, SD)	Number of Days on Sick-Leave per Patient in 12 Months after Prescription (Mean, SD)	Difference in Number of Days on Sick-Leave (95% CI)	*p* Value *
All patients	0.19 (1.25)	0.10 (0.70)	0.09 (0.01–0.16)	0.023
Age 18–30 years	0.15 (0.76)	0.08 (0.56)	0.07 (−0.04–0.17)	0.069
Age 31–40 years	0.18 (0.96)	0.10 (0.74)	0.08 (−0.06–0.22)	0.187
Age 41–50 years	0.20 (0.12)	0.15 (1.02)	0.04 (−0.12–0.21)	0.262
Age 51–60 years	0.23 (1.76)	0.07 (0.48)	0.16 (−0.02–0.33)	0.158
Age 61–70 years	-	-	-	-
Age > 70 years	-	-	-	-
Women	0.19 (1.26)	0.09 (0.70)	0.09 (0.01–0.17)	0.019
Men	0.20 (1.08)	0.14 (0.72)	0.06 (−0.23–0.35)	0.492

SD: standard deviation; CI: confidence intervals; UTI: uncomplicated urinary tract infections. * Wilcoxon test for paired samples.

## Data Availability

The data are available from the corresponding author.
