# Correction: Kostev, K.; Cai, T. Cystitis and Utipro^®^ Plus: Real-World Evidence. *Healthcare* 2023, *11*, 2564

**DOI:** 10.3390/healthcare12010108

**Published:** 2024-01-02

**Authors:** Karel Kostev, Tommaso Cai

**Affiliations:** 1 Epidemiology, IQVIA S.L., 60549 Frankfurt am Main, Germany; 2 Department of Gynecology and Obstetrics, University Hospital Marburg, Philipps-University Marburg, 35043 Marburg, Germany; 3 Department of Urology, Santa Chiara Hospital, 38122 Trento, Italy; ktommy@libero.it; 4 Institute of Clinical Medicine, University of Oslo, 0318 Oslo, Norway

In the original publication [1], there was a mistake in Table 4 as published. The column titles presented in Table 4 were identical to the titles in Table 3. Furthermore, we would like to bring to attention that adjustments were made to the data presented in the last four rows of the table. This was necessitated due to an error that occurred during the writing process. We would like to highlight that the data in the first two rows coincided with the data in the last two rows. Subsequently, the correct data has been provided in order to align with the text and ensure accuracy and consistency. No patients were found in the age groups of 61–70 and >70 years. The corrected Table 4 appears below. The authors apologize for any inconvenience caused and state that the scientific conclusions are unaffected. The original publication has also been updated.

## Figures and Tables

**Table 4:** Changes in the mean number of sick-leave days per patient within 12 months after the prescription of Utipro^®^ Plus versus within 12 months prior to the prescription of Utipro^®^ Plus.

Variable	Number of Days on Sick-Leave per Patient in 12 Months Prior to Prescription (Mean, SD)	Number of Days on Sick-Leave per Patient in 12 Months after Prescription (Mean, SD)	Difference in Number of Days on Sick-Leave (95% CI)	*p* Value *
All patients	0.19 (1.25)	0.10 (0.70)	0.09 (0.01–0.16)	0.023
Age 18–30 years	0.15 (0.76)	0.08 (0.56)	0.07 (−0.04–0.17)	0.069
Age 31–40 years	0.18 (0.96)	0.10 (0.74)	0.08 (−0.06–0.22)	0.187
Age 41–50 years	0.20 (0.12)	0.15 (1.02)	0.04 (−0.12–0.21)	0.262
Age 51–60 years	0.23 (1.76)	0.07 (0.48)	0.16 (−0.02–0.33)	0.158
Age 61–70 years	-	-	-	-
Age > 70 years	-	-	-	-
Women	0.19 (1.26)	0.09 (0.70)	0.09 (0.01–0.17)	0.019
Men	0.20 (1.08)	0.14 (0.72)	0.06 (−0.23–0.35)	0.492

SD: standard deviation; CI: confidence intervals; UTI: uncomplicated urinary tract infections. * Wilcoxon test for paired samples.
